# Urinary, Circulating, and Tissue Biomonitoring Studies Indicate Widespread Exposure to Bisphenol A

**DOI:** 10.1289/ehp.0901716

**Published:** 2010-03-24

**Authors:** Laura N. Vandenberg, Ibrahim Chahoud, Jerrold J. Heindel, Vasantha Padmanabhan, Francisco J.R. Paumgartten, Gilbert Schoenfelder

**Affiliations:** 1 Tufts Center for Regenerative and Developmental Biology and; 2 Department of Biology, Tufts University, Medford, Massachusetts, USA; 3 Institut für Klinische Pharmakologie und Toxikologie, Charité Universitätsmedizin Berlin, Campus Benjamin Franklin, Berlin, Germany; 4 Division of Extramural Research and Training, National Institute of Environmental Health Sciences, National Institutes of Health, Department of Health and Human Services, Research Triangle Park, North Carolina, USA; 5 Department of Pediatrics; 6 Department of Obstetrics and Gynecology and; 7 Department of Molecular and Integrative Physiology, University of Michigan, Ann Arbor, Michigan, USA; 8 Laboratory of Environmental Toxicology, National School of Public Health, Oswaldo Cruz Foundation, Rio de Janeiro, Brazil; 9 Institute of Pharmacology and Toxicology, University of Wuerzburg, Wuerzburg, Germany

**Keywords:** endocrine disruptor, human exposure, PBPK/PBTK model, pregnancy, risk assessment, toxicokinetics

## Abstract

**Background:**

Bisphenol A (BPA) is one of the highest-volume chemicals produced worldwide, and human exposure to BPA is thought to be ubiquitous. Thus, there are concerns that the amount of BPA to which humans are exposed may cause adverse health effects. Importantly, results from a large number of biomonitoring studies are at odds with the results from two toxicokinetic studies.

**Objective:**

We examined several possibilities for why biomonitoring and toxicokinetic studies could come to seemingly conflicting conclusions.

**Data sources:**

We examined > 80 published human biomonitoring studies that measured BPA concentrations in human tissues, urine, blood, and other fluids, along with two toxicokinetic studies of human BPA metabolism.

**Data extraction and synthesis:**

The > 80 biomonitoring studies examined included measurements in thousands of individuals from several different countries, and these studies overwhelmingly detected BPA in individual adults, adolescents, and children. Unconjugated BPA was routinely detected in blood (in the nanograms per milliliter range), and conjugated BPA was routinely detected in the vast majority of urine samples (also in the nanograms per milliliter range). In stark contrast, toxicokinetic studies proposed that humans are not internally exposed to BPA. Some regulatory agencies have relied solely on these toxicokinetic models in their risk assessments.

**Conclusions:**

Available data from biomonitoring studies clearly indicate that the general population is exposed to BPA and is at risk from internal exposure to unconjugated BPA. The two toxicokinetic studies that suggested human BPA exposure is negligible have significant deficiencies, are directly contradicted by hypothesis-driven studies, and are therefore not reliable for risk assessment purposes.

Bisphenol A (BPA) is one of the highest volume chemicals produced worldwide, with > 8 billion pounds produced each year and > 100 tons released into the atmosphere by yearly production. Data from multiple sources indicate that the amount of BPA to which humans are exposed may cause adverse health effects; this has raised concerns among regulatory agencies all over the world.

As an essential component of polycarbonate plastic, BPA is found in numerous consumer products, including baby bottles, reusable water bottles, reusable food containers, polyvinyl chloride stretch films, papers, and cardboards (reviewed by Vandenberg et al. 2007). Metallic food and beverage cans are protected from rusting and corrosion by the application of epoxy resins as inner coatings. The synthesis of many of these resins requires the condensation of BPA with epichlorohydrin to create BPA diglycidyl ether. When incomplete polymerization occurs, residual BPA leaches from the epoxy resin. High temperatures and exposure to acidic or basic solutions can also increase leaching of BPA from coatings and plastics, even when complete polymerization has occurred. More than 10 studies have detected BPA leaching from the linings of metal cans into foods (Vandenberg et al. 2007). BPA has also been detected in a variety of environmental samples, including water, sewage leachates, indoor and outdoor air samples, and dust (Vandenberg et al. 2007). It is also found in papers and in implanted medical devices and other medical equipment (Welshons et al. 2006).

BPA attracted the attention of regulatory agencies and scientists in dozens of countries because of its estrogenic properties *in vitro* and *in vivo* and the conserved role that estrogen plays in regulating human and animal physiology and pathophysiology (Dodds and Lawson 1936; Markey et al. 2001; Wetherill et al. 2007). Biochemical assays have examined the kinetics of BPA binding to the estrogen receptors (ERs) and have determined that BPA binds both ERα and ERβ, with approximately 10 times higher affinity for ERβ (Gould et al. 1998; Kuiper et al. 1998). Until recently, BPA was considered a weak environmental estrogen because of its relatively low affinity for the nuclear ERs compared with estradiol in some assays (Andersen et al. 1999; Fang et al. 2000). However, results from several studies have revealed that BPA can stimulate rapid cellular responses at very low concentrations, below the levels where BPA is expected to bind to the classical nuclear ERs (Welshons et al. 2006). BPA has also been shown to bind to a membrane-associated ER and produce nongenomic steroid actions (Wetherill et al. 2007) with the same efficacy and potency as estradiol (Alonso-Magdalena et al. 2005; Hugo et al. 2008). Whatever the mechanism, BPA can cause effects in animal models at doses in the range of human exposures, indicating that it can act at lower doses than predicted from some *in vitro* and *in vivo* assays (Richter et al. 2007; Vandenberg et al. 2007; vom Saal et al. 2007; Wetherill et al. 2007).

For risk assessment, a reference dose (RfD) is calculated as an acceptable daily human intake, typically 100-fold less than the no observed adverse effect level (NOAEL). However, the RfD for BPA (50 μg/kg/day) was calculated using the lowest observable adverse effect level (LOAEL) and 1,000-fold safety factors because a NOAEL had not been determined (Welshons et al. 2003). More than 150 published studies describe BPA effects in animals exposed to < 50 mg/kg/day, including altered development of the male and female reproductive tracts, organization of sexually dimorphic circuits in the hypothalamus, onset of estrus cyclicity and earlier puberty, altered body weight, altered organization of the mammary gland, and cancers of the mammary gland and prostate; > 40 of these studies examined doses less than the RfD (Richter et al. 2007). Many of these end points are in areas of current concern for human epidemiological trends (Soto et al. 2008; Vandenberg et al. 2009). Indeed, it has been suggested that exposure to xenoestrogens such as BPA during early development may be a major contributing factor to the increased incidence of infertility, genital tract abnormalities, obesity, attention deficit hyperactivity disorder, infertility, and prostate and breast cancer observed in European and U.S. human populations over the last 50 years (Sharpe and Skakkebaek 1993; Soto et al. 2008).

There is great concern about exposure of human fetuses, infants, and neonates to BPA because of the sensitivity of the developing organs and brain to exogenous hormones (Vandenberg et al. 2009). However, for translating the findings from animal studies to health risks of environmental exposures to humans, exposure assessment from biomonitoring of BPA in different populations is essential.

In this review, we examined > 80 biomonitoring studies that measured BPA concentrations in human tissues and fluids, specifically focusing on individuals that were exposed to BPA via their environment (i.e., nonoccupational exposures). These studies, examining thousands of individuals from several different countries, overwhelmingly detected BPA in individual adults, adolescents, and children. Interestingly, results from the large body of research encompassing biomonitoring studies are at odds with the results from two toxicokinetic studies that determined the disposition of BPA in humans after oral administration of BPA (Volkel et al. 2002, 2005).

Volkel et al. (2002) reported that after oral absorption, BPA was promptly metabolized in the liver and intestines. However, findings from the > 80 biomonitoring studies we reviewed support the hypothesis that hepatic metabolism of BPA or its presystemic clearance is not 100% efficient, because unconjugated BPA has been regularly detected in urine and blood samples. Theoretically, the presence of unconjugated BPA in blood and/or urine samples could also be explained by exposure via non-oral routes that circumvent liver and intestinal first-pass metabolism (Stahlhut et al. 2009). All sources for oral and non-oral exposures have not been definitively identified, thus increasing the value of biomonitoring studies, which do not take into account exposure sources. Throughout this review, when we discuss conjugated BPA it is specifically identified; otherwise, “BPA” refers to the unconjugated molecule. Note also that when we identify significant differences, we are referring to statistically significant differences identified by the original study authors, each of whom established a relevant α-value for their study populations.

In this review, we propose several hypotheses to explain why biomonitoring and toxicokinetic studies could come to seemingly conflicting conclusions. Additionally, the reliance of regulatory agencies on the two studies that predict no human exposure, in contrast to the > 80 studies that measure actual internal exposures, is contrary to scientific principles.

## Biomonitoring Studies: General Overview

Biomonitoring studies allow the determination of internal circulating levels and excreted concentrations of a chemical of interest, which account for exposures from all possible sources, rather than suspected exposures from specific sources. In the United States, the Centers for Disease Control and Prevention (CDC) is the major source for information on human exposures to a multitude of environmental contaminants and has developed sensitive assays to reliably measure them (CDC 2008; Kuklenyik et al. 2009). In Europe, the German Environmental Survey is a representative population study to determine the exposure of Germany’s general population to environmental contaminants (Umwelt Bundes Amt 2009). Its main objective is to generate, update, and evaluate representative data in order to facilitate environmental health–related observations and reporting of information at the national level.

Proper biomonitoring studies take into account the kinetics, bioaccumulative properties, and metabolism of the target chemical to determine which tissue (biological matrix) should be examined. The chemical properties of the substance being examined also have an impact on the matrices that can be examined reliably, because some substances are altered by enzymes in blood and other substances can break down in urine (Calafat and Needham 2008). Furthermore, the toxicokinetics of the substance being investigated is likely to be influenced by the physiological status of the individual. Finally, the sensitivity and reliability of the methods used for analysis, the collection methods and materials, and the contamination of laboratory chemicals and equipment with the substance of interest are all important factors to be considered in biomonitoring studies. We discuss these issues in greater detail below.

## Analytical Methods Used in Biomonitoring Studies

Multiple techniques have been used to measure total, unconjugated, and conjugated BPA in human blood, urine, and tissue samples. Gas chromatography (GC) and liquid chromatography (LC) are typically used with various detection methods, including mass spectrometry (MS), tandem MS (MS/MS), and electrochemical or fluorescence derivatization. The CDC has measured BPA using solid-phase extraction coupled with isotope dilution–HPLC (high-performance liquid chromatography)–MS/MS, which is considered the “gold standard” for urine biomonitoring studies because of its high level of accuracy, negligible interference, and ability to identify chemical structures (Calafat et al. 2008). However, this method is limited by its high cost per sample, making it impractical for many studies.

The methods used in biomonitoring studies are crucial for the acceptance of the results (Dekant and Volkel 2008; Vandenberg et al. 2007). The ELISA (enzyme-linked immunosorbent assay) has also been used in several studies of human fluids and tissues because it is convenient, inexpensive, and useful for the screening of a large number of samples. However, the use of ELISA to measure BPA concentrations in human samples has been specifically challenged because this method is considered less specific than methods employing analytical chemistry (Dekant and Volkel 2008; Fukata et al. 2006); that is, there is concern that ELISA assays detect substances other than BPA and its conjugates, including other bisphenols (Ohkuma et al. 2002). Studies rarely report the controls that are necessary to determine whether or not cross-reactivity has occurred; however, if information about cross-reactivity and standard validations are included, these data should be considered valid, as they are for the many other compounds that are measured with this method. In general, ELISA has fallen out of favor for the quantification of BPA in human biomonitoring studies, particularly because more sensitive, accurate, and precise methods employing analytical chemistry are now available for high-throughput screens (Kuklenyik et al. 2009). Nonetheless, it should be recognized that this method is much more affordable than analytical chemistry methods and, most important, it has successfully been used to measure BPA in samples in a comparable dose range and in a similar percentage of samples as the methods that are considered more accurate (Vandenberg et al. 2007).

In addition to the methods used to measure BPA in human tissues and fluids, the equipment and containers used to collect and store samples are critical for the accurate assessment of BPA concentrations. It has been suggested that the low levels of unconjugated BPA detected in bodily tissues and fluids were due to contamination from collection materials or nonenzymatic deconjugation of BPA during storage (Atkinson et al. 2002; Dekant and Volkel 2008; Willhite et al. 2008). Organic solvents used in the laboratory may release BPA from plastic labware, leading to contamination; water has also been found to contain detectable levels of BPA (Vandenberg et al. 2007), and the columns used for HPLC may contain and/or trap BPA (Volkel et al. 2005). For these reasons, the use of adequate blanks is essential for every step of the biomonitoring process, including sample collection and every point in the chemical analysis. These blanks should be subjected to the same extraction/injection protocols as the actual samples. Most of the biomonitoring studies we reviewed included data from blanks to indicate that, contrary to the claims made by some scientists (Dekant and Volkel 2008), contamination from equipment cannot explain the concentrations of BPA reported.

In terms of sample collection, studies should control for contamination from syringes used to draw samples, pipettes used to transfer plasma/serum to storage tubes, and the nature of sample storage tubes. If samples are obtained in hospital settings where the patient has an intravenous tube, it is essential to account for BPA leaching from these tubes during saline infusion. Studies need to be performed using appropriate matrix (blood or urine) spiked with known amounts of BPA and subjected to collection procedures similar to that of actual sampling to rule out BPA contribution from these various sources. One study used sheep blood to perform such validations and found very little BPA contamination from these sources (Padmanabhan et al. 2008). However, for translation to humans, similar studies using human blood and/or urine need to be undertaken.

Ye et al. (2007) addressed the issue of sample storage by comparing the stability of BPA conjugates for urine stored for up to 6 months at three temperatures: room temperature, 4°C, or −70°C. Storage at −70°C kept conjugated BPA stable for at least 180 days, whereas storage at room temperature led to degradation of BPA conjugates within 24 hr. The effects of storage temperature on the detection of BPA and BPA conjugates in saliva samples have also been investigated. Atkinson et al. (2002) spiked saliva samples with BPA, BPA dimethylacrylate (Bis-DMA), or triethylene glycol dimethacrylate (TEGDMA), stored at −20°C or −70°C, and then tested the samples by HPLC and GC-MS. In contrast to samples stored at −20°C, concentrations of BPA, Bis-DMA, and TEGDMA were unchanged in salivary samples stored at −70°C, suggesting that this condition was appropriate for biological sample storage. Finally, in a recent study Ye et al. (2009b) examined the stability of phenols in 16 commercially available serum samples stored under worst-case–scenario conditions (4°C, 25°C, or 37°C). Concentrations of BPA metabolites and other phenols did not vary significantly, even when samples were stored at 37°C for at least 30 days. This finding suggests that phenolic compounds, likely including BPA, are more stable in serum than in urine, and it gives greater credence to those studies that measured BPA in human serum samples.

## Exposure Assessment from Urinary Measures of BPA

In 2001, the CDC conducted the first biomonitoring study of BPA (unconjugated and conjugated) in pooled urine samples (Brock et al. 2001). The authors developed a method that was fairly sensitive [limit of detection (LOD) = 0.12 ng/mL]; concentrations of unconjugated BPA were < LOD, and concentrations of BPA glucuronide ranged from 0.11 to 0.51 ng/mL. After this initial examination in pooled urine, more than a dozen additional small studies examined urinary BPA concentrations and/or its metabolites in < 100 adults each (Arakawa et al. 2004; Carwile et al. 2009; García-Prieto et al. 2008; Joskow et al. 2006; Kawaguichi et al. 2005; Kim et al. 2003; Liu et al. 2005; Mao et al. 2004; Matsumoto et al. 2003; Moors et al. 2007; Nepomnaschy et al. 2009; Ouichi and Watanabe 2002; Schöringhumer and Cichna-Markl 2007; Tsukioka et al. 2003; Volkel et al. 2005; Yang et al. 2003; Ye et al. 2005a, 2005b) (Table 1). Although these studies used slightly different methods and different population samples, they overwhelmingly detected BPA or its conjugates in urine. Collectively, these studies examined spot urine samples collected from 604 adults, and BPA and/or its conjugates were detected in 518 (85.8%).

Because most studies of urine used enzymatic treatment with either glucuronidase or sulfatase, or both, many studies reported only the total BPA concentration, that is, unconjugated plus conjugated BPA. Of the seven studies that specifically examined unconjugated BPA, six detected unconjugated BPA in at least one sample (Calafat et al. 2009; Kim et al. 2003; Ouichi and Watanabe 2002; Schöringhumer and Cichna-Markl 2007; Volkel et al. 2008; Ye et al. 2005b); only one study failed to detect unconjugated BPA in any sample (Brock et al. 2001). The distinction between conjugated and unconjugated BPA is an important one, especially in blood samples, where the presence of unconjugated BPA in circulation indicates internal exposure to the parent compound, which is estrogenically active. The presence of unconjugated BPA in urine is also indicative of internal exposure to BPA and suggests either a failure of first-pass metabolism to conjugate and rapidly remove BPA from the body or the deconjugation of BPA metabolites in the body. It is also possible that these repeated measurements of unconjugated BPA in urine are the result of nonenzymatic deconjugation of conjugated BPA during storage, although storage protocols provided in these studies and its repeated detection suggest that this is unlikely.

In contrast to the many small studies detecting BPA and/or its conjugates in urine, one study failed to detect either unconjugated or conjugated BPA in the 19 samples examined (Volkel et al. 2005). To our knowledge, this is the only published study that completely failed to detect or measure any form of BPA in urine samples from environmentally exposed individuals. Volkel et al. (2005) used HPLC-MS/MS; this is the same technique that was used in several other studies in which unconjugated and conjugated BPA were detected in urine (Calafat et al. 2008; Hong et al. 2009; Mahalingaiah et al. 2008; Nepomnaschy et al. 2009; Teitelbaum et al. 2008; Wolff et al. 2007; Yang YJ et al. 2009; Ye et al. 2009a). Why did Volkel et al. (2005) not detect any form of BPA? The first possibility to consider is whether their study was the only one that successfully avoided external contamination of samples with BPA. Because several of these biomonitoring studies were performed by high-standard analytical laboratories (e.g., CDC), it is unlikely that all of these studies except the one by Volkel et al. (2005) failed to adequately prevent external contaminations (Ginsberg and Rice 2009). The second possibility to consider is whether Volkel et al. (2005) used a method with sufficient sensitivity to detect any form of BPA in urine. Unlike the other studies using HPLC-MS/MS, which is generally a very sensitive method (LODs ≤ 0.4 ng/mL), the LOD of Volkel et al.’s study was 1.14 ng/mL. Two other studies used analytical methods with similar or higher LODs: Matsumoto et al. (2003) used HPLC and detected total BPA in most of the 106 individuals examined (LOD = 1.7 ng/mL), and Mao et al. (2004) used HPLC with fluorescence detection and found total BPA in most of the 20 individuals examined (LOD = 2.7 ng/mL). In addition, considering the mean (or median) concentrations of total BPA detected in other studies examining urine samples with HPLC-MS/MS [2.0 ng/mL (Wolff et al. 2007); 2.6 ng/mL (Calafat et al. 2008); 1.3 ng/mL (Mahalingaiah et al. 2008); 2.7 ng/mL (Hong et al. 2009); 1.82 ng/mL (Nepomnaschy et al. 2009); 0.52–0.61 ng/mL (Yang YJ et al. 2009); 2.52–4.50 ng/mL (Ye et al. 2009a)], this less sensitive analytical method would likely be able to detect some form of BPA in the average person. To address the issue of whether their experimental system was able to detect BPA at all in urine samples, Volkel et al. (2005) spiked urine samples with large amounts of BPA glucuronide. Under these conditions, they were able to successfully detect this compound using their methodology, suggesting that there were no problems detecting high concentrations of BPA metabolites in urine. Considering that this study examined < 20 adults, the negative result reported by Volkel et al. (2005) could be explained as a sampling error combined with a relatively insensitive analytical method, but this is unlikely, leaving us with no scientific explanation for their lack of detection of BPA. Because science is based on repeatability of data, the fact that this one negative study stands against more than a dozen studies that successfully measured BPA in urine samples suggests that it is an outlier.

The smaller studies described above provide little information about exposure of the general population. In 2005, the first study to measure total BPA concentrations in a reference population was performed by the CDC (Calafat et al. 2005); total BPA was detected in 95% of spot urine samples collected from 394 American adults. The CDC followed this study with a second one, examining spot urine samples from 2,517 Americans > 6 years of age from the 2003–2004 National Health and Nutrition Examination Survey (NHANES) (Calafat et al. 2008). Total BPA was detected in 92.6% of participants, with concentrations ranging from 0.4 to 149 ng/mL and a geometric mean (GM) of 2.6 ng/mL urine.

Urinary volume is influenced by various factors (i.e., glomerular filtration, tubular secretion and reabsorption, alimentary regimen, intake of liquids, perspiration) that give rise to differing urinary dilutions and, consequently, to changes in the concentrations of excreted substances (Carrieri et al. 2000). Dilute or concentrated samples provide low or high values, respectively, of the biological marker, with underestimation or overestimation of the result. Therefore, to compare exposures among groups of individuals, urinary concentrations of chemicals must be adjusted to urinary creatinine concentration (Barr et al. 2005). After correcting for urine creatinine concentration, Calafat et al. (2008) found that total urinary BPA concentrations were associated with age, sex, race/ethnicity, and household income.

Four additional studies examined fairly large populations of adults. In a study of 172 Korean adults, Yang et al. (2006) found that 97.5% had detectable levels of total BPA, and in a study of 516 Korean adults, Hong et al. (2009) found detectable levels of total BPA in 76%. Yang YJ et al. (2009) examined urine from 485 Korean adults (259 men, 92 premenopausal women, and 134 postmenopausal women) and found detectable levels of total BPA in 76%, a strikingly similar result. In the fourth study, Volkel et al. (2008) examined 438 urine samples from 285 individuals, as well as additional samples collected from members of the research team; some of the samples examined had been stored for several years at −20°C. Because of problems with methodology, total BPA could only be measured in a subset of the samples; total BPA concentrations ranged from undetected levels to 9.3 ng/mL. In sum, these six large-scale studies (Calafat et al. 2005, 2008; Hong et al. 2009; Volkel et al. 2008; Yang et al. 2006; Yang YJ et al. 2009) reinforce findings from small studies, providing further evidence that most individuals in several countries have detectable levels of BPA and/or its conjugates in their urine (Table 1).

Levels of BPA exposure and concentrations in bodily fluids and tissues from individuals in less developed countries have been relatively understudied and were previously identified as an important research need (Vandenberg et al. 2007). In a recent study in China, He et al. (2009) addressed this issue by examining > 900 human subjects. Detectable levels of total BPA were measured in 50% of the subjects. With this large sample size, the authors were able to detect several statistically significant associations: Detection rates were higher in males, in people > 40 years of age, in people with more education, and in individuals who smoked and/or consumed alcohol. Some of these results differ from the CDC’s findings in the United States (i.e., sex- and age-related differences in exposure) and thus may suggest that exposure routes or sources are different between these populations.

Biomonitoring measurements for adults are generally not informative about internal concentrations or exposure levels in children. The CDC study (Calafat et al. 2008) examined total BPA content in spot urine samples from 314 American children 6–11 years of age and reported a GM concentration of 3.6 ng/mL urine. The GM level of total BPA in the 715 adolescents 12–19 years of age examined in the same study was 3.7 ng/mL urine. When adjusted for creatinine levels, exposures were highest in children and still significantly higher in adolescents than in adults. Six additional studies measured BPA and its metabolites in urine of infants and children. Liu et al. (2005) examined urine from nine 9-year-old American girls and detected total BPA in eight of the girls. Wolff et al. (2007) detected total BPA in 94% of urine samples collected from 90 American girls 6–9 years of age. In another study, Teitelbaum et al. (2008) examined 159 urine samples that had been collected from 35 black and Hispanic American children 6–10 years of age; of these spot urine samples, 95% had detectable concentrations of total BPA, with a GM of 3.4 ng/mL urine, a level very similar to those of children examined in the larger CDC study (Calafat et al. 2008). In a large study that included a subset of 30 German children 5–6 years of age, Volkel et al. (2008) measured unconjugated BPA concentrations up to 0.9 ng/mL and total BPA concentrations up to 7.5 ng/mL, although the frequency of detection was not reported. In a study of 599 German children 3–14 years of age, Becker et al. (2009) detected total BPA in the urine of 591 individuals (98.7%; GM of 2.66 ng/mL); they detected significantly higher levels in children 3–5 years of age (GM, 3.55; *n* = 137). Finally, Calafat et al. (2009) studied 41 premature infants in an American neonatal infant care unit and found BPA conjugates in the urine of all the babies examined. Amazingly, the GM concentrations measured in these infants were 11 times higher than the concentrations measured in the NHANES study (30.3 vs. 2.6 ng/mL urine). Unconjugated BPA was also detected in the urine of 34 of the 37 neonates examined, with a GM of 1.8 ng/mL urine (Calafat et al. 2009). These authors noted that the presence of unconjugated BPA in urine was unexpected, and because of the collection of urine from cotton placed in diapers, there is uncertainty as to whether this is a contaminant from the collection process or a true measure of unconjugated BPA from these young individuals. Collectively, these seven studies included 41 premature infants (Calafat et al. 2009), 909 children 3–11 years of age (Becker et al. 2009; Calafat et al. 2008; Liu et al. 2005; Teitelbaum et al. 2008; Volkel et al. 2008; Wolff et al. 2007), and 883 adolescents 12–19 years of age (Becker et al. 2009; Calafat et al. 2008) and repeatedly found detectable levels of total BPA in a vast majority of the individuals examined. These studies clearly indicate that children are exposed to BPA and suggest that exposures are highest among neonates and young children.

Four studies examined total BPA concentrations in urine collected from women during pregnancy, further suggesting exposure of the fetus to BPA during gestation (Table 1). First, in a study examining spot urine samples collected from 100 pregnant Dutch women, Ye et al. (2008b) detected total BPA in 82% of samples, with a median concentration of 1.2 ng/mL urine, similar to the levels of BPA detected in other populations, including U.S. reference populations (Calafat et al. 2005, 2008). An even larger study examined urine from 404 American women in their third trimester of pregnancy. In that study Wolff et al. (2008) detected total BPA in the urine of 90.8% of the women, and urine concentrations were associated with offspring birth weight. A third study examined total BPA in 10 pooled urine samples from 110 pregnant Norwegian women (Ye et al. 2009a); the concentrations measured were higher than in any other study of pregnant women (mean, 4.5 ng/mL). These authors also examined urine samples from 110 pregnant Dutch women and 87 pregnant American women. The concentrations in these populations were relatively high compared with other groups that have been examined, with mean concentrations of 2.52 and 3.93 ng/mL, respectively (Ye et al. 2009a). In the final study to include urine samples from pregnant women (Mahalingaiah et al. 2008), samples were collected from a fertility center in Massachusetts. In urine samples collected from 45 women before pregnancy, the GM concentration of total BPA was 1.09 ng/mL urine. During the study period, 10 subjects became pregnant. In these women, total BPA concentrations in their urine were 26% higher than in urine of nonpregnant women and 33% higher than in urine samples collected before pregnancy. This last finding is perhaps the most concerning indicator of exposure of the human fetus to BPA.

Other studies have estimated associations between BPA concentrations and activities that may increase BPA exposure. For example, Matsumoto et al. (2003) associated the urinary concentrations of Japanese subjects with their consumption of coffee and tea drinks from cans containing BPA resins. Interestingly, when can coatings were changed to a formula with a lower BPA content, the association they found was no longer evident; in addition, removal of BPA from can linings led to a > 50% reduction in conjugated BPA detected in urine. Carwile et al. (2009) measured total BPA concentrations in urine collected from a group of American college students during a washout phase when all cold beverages were consumed from stainless steel containers, and compared these concentrations with those from the same students during a phase when all cold beverages were consumed from polycarbonate plastic containers. The authors found that urinary concentrations of total BPA were increased significantly (69% higher) during the week when polycarbonate bottles were used, suggesting that these containers may be a significant source of BPA exposure to individuals in this age group and that interventions would help lower exposure levels. Joskow et al. (2006) made an interesting observation about urinary concentrations of total BPA in male volunteers receiving dental sealant treatments: Compared with baseline levels (2.41 ng/mL), individuals who received one brand of sealant known to contain BPA had significantly more BPA metabolites in their urine 24 hr after sealant placement (7.34 ng/mL), whereas individuals who received a different sealant had concentrations similar to the pretreatment level (2.06 ng/mL) (Joskow et al. 2006). Another brief study examined total BPA in urine samples collected from American children before and after placement of dental sealants (Martin et al. 2005). Total BPA levels increased rapidly from baseline levels (0.26 ng/mL) within 24 hr of sealant placement (1.18 ng/mL) and peaked 7 days after placement (1.21 ng/mL). However, the measured concentrations did not return to baseline 14 days after placement (0.73 ng/mL), suggesting that exposure of children to BPA from dental sealants may be more than the acute exposure that has been suspected. Finally, Schöringhumer and Cichna-Markl (2007) compared urinary concentrations of unconjugated and total BPA in 10 dialysis patients and 12 healthy adults but found no significant differences between these two groups.

## Estimates of BPA Consumption from Urinary Measures

Only a few studies have estimated total BPA exposure in the human population. Using exposure estimates from a variety of environmental sources (i.e., water, air, and soil) and from food and beverage contamination (i.e., leaching rates from plastic containers and can linings), several studies estimated daily human intake of < 1 μg/kg body weight (BW)/day (Kang et al. 2006; Wilson et al. 2003, 2007). Additional studies have estimated daily BPA exposure by calculating BPA ingestion from food sources (European Union 2002). Biomonitoring data provide more reliable information about exposures because they do not require all the sources of exposure to be identified. This is especially important for the case of BPA, where non-oral routes of exposure are suspected and the totality of sources has not yet been identified (Stahlhut et al. 2009; Welshons et al. 2006).

Using toxicokinetics, urinary BPA levels have been extrapolated to estimate daily intake levels. For instance, using urine samples collected from 48 women, Ouichi and Watanabe (2002) estimated daily intake to be 0.6–71.4 μg/day. Using backward calculations from urinary BPA concentrations detected in Japanese adults, worst-case–scenario daily intakes for that population were estimated at 0.037–0.064 μg/kg BW/day for males and 0.043–0.075 μg/kg BW/day for females (Miyamoto and Kotake 2006). Kamrin (2004) utilized urine concentrations reported in two previous studies (Arakawa et al. 2004; Brock et al. 2001) to calculate daily intake levels of 0.002–0.3 μg/kg BW/day. Although these calculations and estimates have provided a range of doses that researchers can target for experimental animal studies, these figures were determined based on toxicokinetic models that may be flawed, a topic that we discuss in further detail below.

## Salivary Measures of BPA

Like urine, saliva is a preferred bodily fluid for biomonitoring purposes because collection requires relatively noninvasive procedures. To date, studies that have examined saliva for BPA have focused on the effects of dental sealant application to BPA concentrations. Since the 1960s, BPA diglycidyl methacrylate has been used as a component of many dental restorative materials, including those used for sealing molars.

Six studies have measured BPA in saliva after dental sealant application, and all were able to detect BPA in the saliva of some of the individuals examined (Arenholt-Bindslev et al. 1999; Fung et al. 2000; Joskow et al. 2006; Olea et al. 1996; Sasaki et al. 2005; Zafra et al. 2002) (Table 2). These studies used different analytical methods and examined saliva collected at different points after sealant application. Although these studies provide interesting information about the dynamics of BPA leaching from sealants shortly after sealant placement (Vandenberg et al. 2007), they are less informative about the use of saliva as a matrix for biomonitoring of BPA. Joskow et al. (2006) measured BPA concentrations in saliva before any treatment, with a mean level of 0.3 ng/mL saliva; this concentration is much lower than that measured in urine (Table 1). Owing to the possibility of contamination with BPA leaching from dental materials, saliva does not seem to be a reliable biomonitoring tool for estimating systemic exposure to unconjugated BPA.

## Internal Dosage Assessment from Blood Measures of BPA

Seventeen studies have measured BPA in blood and serum samples from healthy male and nonpregnant female adults (Dirtu et al. 2008; Fung et al. 2000; He et al. 2009; Hiroi et al. 2004; Ikezuki et al. 2002; Inoue et al. 2000, 2001; Kaddar et al. 2009; Kuroda et al. 2003; Liu et al. 2007; Sajiki et al. 1999; Sugiura-Ogasawara et al. 2005; Takeuchi and Tsutsumi 2002; Takeuchi et al. 2004; Volkel et al. 2005; Yang M et al. 2009; Yoshimura et al. 2002) (Table 3). Of these studies, five used ELISA, one used a radioimmunoassay (RIA), and the remainder used analytical chemistry to assess BPA concentrations. These studies had similar LODs compared with studies of urine, but unlike most urine studies, measurements performed on blood, serum, or plasma samples usually measured unconjugated BPA specifically. In healthy, nonpregnant adults, unconjugated BPA was detected in 14 of 16 studies (including 8 of 10 studies using analytical chemistry). Compared with the magnitude of studies examining BPA levels in urine, most of these studies involved smaller sample sizes. Large-scale biomonitoring studies measuring circulating levels of unconjugated BPA, an index of internal exposure levels, remain to be done, particularly in the United States; only two studies to date examined > 100 individuals, and neither of these included samples from Americans. However, of those studies that used analytical chemistry methods and measured detectable levels of BPA, mean concentrations were typically in the range of 1 ng/mL blood. These concentrations are quite similar to those reported for BPA conjugates in urine (Table 1) and clearly indicate that humans are internally exposed to unconjugated BPA.

In one of the larger studies of human blood, Kaddar et al. (2009) examined plasma samples from 207 individuals, collected randomly in a French hospital, using an RIA (LOD = 0.08 ng/mL plasma; correlation with HPLC-MS, *r*^2^ = 0.92). Unconjugated BPA was detected in 83% of the samples, and 12% had > 2 ng/mL plasma. Patients undergoing dialysis were also examined in this study, and > 70% of patients had measurable concentrations of unconjugated BPA > 10 ng/mL plasma. In another recent study performed in China, He et al. (2009) measured total BPA concentrations in > 900 individuals. Because samples were treated with β-glucuronidase, it is impossible to determine the proportion of conjugated and unconjugated BPA that was present. Measurable levels of total BPA were found in 17% of the samples; detection levels were significantly higher in females than in males and in people < 40 years of age compared with people > 40 years of age, and were highest in individuals who smoked and/or consumed alcohol. Finally, a study examining stored blood samples collected from Korean women during 1994–1997 measured both unconjugated and total BPA in > 150 individuals (Yang M et al. 2009). About half of these women were breast cancer patients, and the other half were age-matched controls. These authors found no associations between breast cancer status and BPA concentrations. Total BPA was detected in 50.8% of the samples. However, the levels of unconjugated BPA in most samples were < LOD (0.012 ng/mL).

We are aware of only two studies that were unable to detect BPA in any individual samples of blood from adults (Fung et al. 2000; Volkel et al. 2005). Fung et al. (2000) examined 40 adults before and after the application of dental sealant materials containing BPA. This study had an LOD of 5 ng/mL; considering other studies that suggest exposures are typically in the 1 ng/mL range, it is not surprising that this study was unable to detect BPA in blood. In the second study that did not detect BPA in the blood of healthy individuals, Volkel et al. (2005) used LC-MS/MS, typically a highly sensitive method, but examined only 19 adults. The authors of that study reported multiple LODs for blood samples (0.57 and 1.14 ng/mL), although a later study from this group suggested that the LOD was 0.5 ng/mL blood (Dekant and Volkel 2008). That study, which examined only 19 adults, was the same one that was unable to detect any form of BPA in urine (Volkel et al. 2005), suggesting that it is hindered by methodological problems.

Several studies that examined BPA concentrations in blood or serum from relatively healthy adults also measured BPA in individuals with diseases or health-related conditions. Two studies found women with polycystic ovarian syndrome (PCOS) had higher serum levels of BPA than did healthy control women (Takeuchi and Tsutsumi 2002; Takeuchi et al. 2004). These studies also found a positive association between serum testosterone levels and BPA concentrations; this finding is especially interesting because it provides a potential basis for gender-biased exposures or metabolism of BPA (Takeuchi et al. 2006).

Because of concerns that BPA alters the development of rodents exposed during gestation, several human biomonitoring studies have focused on measuring BPA in serum from pregnant women, and in plasma, serum, and tissue from umbilical cords (Ikezuki et al. 2002; Kuroda et al. 2003; Lee et al. 2008; Padmanabhan et al. 2008; Schönfelder et al. 2002; Tan and Ali Mohd 2003; Todaka and Mori 2002; Yamada et al. 2002) (Tables 4 and 5). Of the eight studies examining these populations, six used analytical chemistry and two used ELISA. Regardless of the method used for detection, every study was able to detect unconjugated BPA in at least some of the samples collected. Interestingly, the levels detected in pregnant women were typically higher than those reported for nonpregnant adults; several studies that used analytical chemistry to assess BPA concentrations measured mean concentrations in the blood or serum from pregnant women at > 4 ng/mL (Lee et al. 2008; Padmanabhan et al. 2008; Schönfelder et al. 2002). However, only two studies collected samples from pregnant and nonpregnant women and directly compared them using identical methods; one reported slightly lower levels in pregnant women (Ikezuki et al. 2002), and the other found no differences in these two populations (Kuroda et al. 2003). Thus, this is also an important area for future research.

Of the four studies that measured unconjugated BPA in umbilical cord blood (Table 4), two detected levels higher in fetal blood than in maternal samples (Ikezuki et al. 2002; Kuroda et al. 2003), and two detected levels higher in maternal samples than in fetal blood (Lee et al. 2008; Schönfelder et al. 2002). Interestingly, two of these studies detected concentrations in a range (~ 1 ng/mL) similar to those measured in healthy adults (Kuroda et al. 2003; Lee et al. 2008). The other two studies measured BPA concentrations two to four times higher in fetuses than in nonpregnant women (Ikezuki et al. 2002; Schönfelder et al. 2002). As with the maternal blood samples, only two studies directly compared plasma/blood BPA concentrations in fetuses and nonpregnant adults (Ikezuki et al. 2002; Kuroda et al. 2003), so it is difficult to make any conclusive comparisons regarding internal concentrations, metabolism, or exposure.

With the exception of one recent study that measured BPA concentrations in the blood of 300 women and their fetuses (Lee et al. 2008), the studies mentioned above are limited because of their relatively small sample sizes. This one large-scale study (Lee et al. 2008) is the most robust blood biomonitoring study of BPA to date. Despite the relatively high LOD (0.625 ng/mL) for the method used, total BPA was detected in the blood of 84% of pregnant women and 40% of fetal samples collected; 23% of blood samples collected from pregnant women contained total BPA concentrations > 10 ng/mL, but concentrations of unconjugated BPA alone were not measured.

The overall consensus that can be determined from blood sampling of healthy adults, adults with certain diseases, pregnant women, and fetuses is that internal exposures to unconjugated BPA are in the range of 0.5–10 ng/mL, with most studies suggesting an average internal exposure of approximately 1–3 ng/mL (Vandenberg et al. 2007). These concentrations are higher than those required to stimulate responses in cell cultures (Wetherill et al. 2007), suggesting that these low levels could influence biological end points and development in humans (vom Saal et al. 2007). At this time, no information is available on BPA concentrations in the blood of infants after birth or of children and adolescents, indicating a significant data gap. Because neonates are not thought to have the metabolic enzymes to effectively conjugate BPA (Mykkanen et al. 1997; Taylor et al. 2008), it is plausible and even likely that these individuals have higher concentrations of unconjugated BPA in their blood than do adults (de Wildt et al. 1999).

## BPA Measurements in Amniotic Fluid, Placenta, and Follicular Fluid

Only a few studies have examined additional tissues and fluids associated with pregnancy (Table 5). In 2002, Schönfelder et al. (2002) measured unconjugated BPA concentrations in placental tissue from 37 pregnancies. BPA was detected in all samples, ranging from 1.0 to 104.9 ng/g tissue, with a median value of 12.7 ng/g and a mean value of 11.2 ng/g. This study suggests that BPA is transplacentally transferred to the embryo/fetal compartment. Additional studies support the idea that the fetus is continuously exposed to BPA; three studies have demonstrated that it can be measured in amniotic fluid (Engel et al. 2006; Ikezuki et al. 2002; Yamada et al. 2002). Ikezuki et al. (2002) found eight times higher concentrations of unconjugated BPA in amniotic fluid from early pregnancy compared with later pregnancy; they proposed that BPA may accumulate in early fetuses because of a lower metabolic clearance of BPA or may be conjugated more efficiently in the fetal liver during later gestation. In another study, Engel et al. (2006) detected unconjugated BPA in < 10% of the amniotic fluid samples collected from pregnancies before 20 weeks of gestation. Collectively, these studies lend support to the idea that the fetus is exposed to unconjugated BPA. In summary, additional studies using larger sample sizes, collection of samples controlled for BPA contamination, and sensitive methods of detection are needed to more accurately quantify BPA in amniotic fluid and placental tissue. However, published studies indicate that the fetus is likely exposed to unconjugated BPA via maternal uptake.

With an increase in the number of successful pregnancies that have resulted from assisted reproductive technologies, two studies examining unconjugated BPA levels in follicular fluid raise additional concerns (Ikezuki et al. 2002; Kaddar et al. 2009) (Table 5). First, Ikezuki et al. (2002) found an average concentration of 2.4 ng/mL fluid collected from women undergoing *in vitro* fertilization (IVF) procedures. Second, Kaddar et al. (2009) measured BPA in follicular fluid from 28 infertile women undergoing IVF. The authors detected unconjugated BPA in 11 of these samples (39%) at concentrations that ranged from 0.15 to 1 ng/mL fluid. These studies have several limitations, including the analytical methods used and the selection of human subjects. However, studies from rodents suggest that BPA can cause aneuploidy in oocytes (Susiarjo et al. 2007) and alter the body weight of offspring resulting from intrauterine transplantation of embryos cultured in media containing BPA (Takai et al. 2001). As such, presence of measurable concentrations of unconjugated BPA in follicular fluid is a potential concern.

## BPA Exposure Assessment in Breast Milk

There is significant interest in defining all sources of BPA exposure, especially those specific to neonates, infants, and children; baby bottles and packaging of infant formulas are currently identified sources of oral BPA exposure (Gies et al. 2009; Vandenberg et al. 2007). An additional and important consideration for the health of the developing neonate is potential BPA exposure from breast milk. BPA is somewhat lipophilic [*K*_ow_ (octanol–water partitioning coefficient) = 2.2–3.4], allowing it to partition into fat and breast milk. Four small studies using analytical chemistry have measured BPA in the breast milk of healthy women (Table 5). The first study examined breast milk from three women and found detectable levels of unconjugated BPA in two of the samples (Otaka et al. 2003). In another study, Sun et al. (2004) detected unconjugated BPA (range, 0.28–0.97 ng/mL; mean, 0.61 ng/mL) in the breast milk of 100% of the women studied. A third study detected unconjugated BPA in 60% of samples (mean, 1.3 ng/mL milk) and total BPA in 90% (mean, 1.1 ng/mL milk) (Ye et al. 2006). In the fourth study, Ye et al. (2008a) detected unconjugated and total BPA in all four of the samples examined.

In a larger study, Kuruto-Niwa et al. (2007) examined BPA concentrations in human colostrum, the milk produced within the first 3 days after giving birth that contains high levels of antibodies, carbohydrates, and protein. Using ELISA, the authors reported detecting unconjugated BPA in 100% of the 101 samples examined (range, 1–7 ng/mL colostrum; mean, 3.41 ng/mL). Although the sample sizes in these studies are relatively small, these findings highlight the concerns about exposure of human infants to BPA via breast milk in addition to other exposures from baby bottles and other containers.

## BPA Exposure Assessment in Adipose Tissue

To address the potential for BPA to accumulate in adipose tissue, Fernandez et al. (2007) examined 20 adult women from Spain for both unconjugated BPA and chlorinated BPA derivatives. Chlorinated BPA derivatives are thought to form when BPA is conjugated in treated water sources. Fernandez et al. (2007) detected unconjugated BPA in 55% of the samples and Cl_2_BPA in 80% of samples; other chlorinated derivatives, including ClBPA and Cl_3_BPA, were detected less frequently, and Cl_4_BPA was not detected in any sample. Of those samples in which BPA was detected, concentrations ranged from 1.80 to 12.01 ng/g adipose tissue (mean, 3.16 ng/g) (Fernandez et al. 2007). In another study, Nunez et al. (2001) reported that repeated exposure to relatively high BPA doses resulted in the distribution of BPA to bodily tissues in rats, including adipose tissue. Human biomonitoring studies using adipose tissue are understandably quite limited because of the invasive procedures needed to isolate this biological matrix, so it is not likely that this study will be repeated with a larger sample size or a broader population. However, it does point out that there are other metabolites of BPA that should be examined for biological and hormonal activity.

## Relationships between Measured BPA Concentrations and Disease Outcomes

In 2008, a large and well-controlled study of the possible health effects of BPA exposure on humans was conducted using samples and information collected for NHANES (Lang et al. 2008). By examining data from 1,455 American adults, these authors found positive associations between urinary (total) BPA concentrations and the prevalence of diabetes, heart disease, and liver toxicity. In a recent study, Melzer et al. (2010) retested the originally identified associations between higher urinary total BPA concentrations and reported heart disease using a second NHANES database. Indeed, the authors were able to replicate earlier associations that were detected between higher urinary concentrations of total BPA and an increased prevalence of coronary heart disease, even though urinary BPA concentrations in this second study were substantially lower than those measured in the first (Lang et al. 2008). In both of these studies, adult exposure levels were associated with chronic diseases that probably began much earlier in the individual’s life. Also of note is that, as with most epidemiological studies, it is impossible to determine cause-and-effect relationships between measured concentrations and disease states. Therefore, the authors cannot determine whether higher concentrations of BPA cause these conditions, or if the presence of these diseases leads to increased exposure or decreased metabolism of BPA; either of these situations is potentially concerning. Additional studies are therefore needed to determine whether there is a causal link between elevated BPA concentrations in urine and these chronic diseases. In addition, it should be noted that these studies used only single urine collections from the individuals examined (Lang et al. 2008; Melzer et al. 2010); multiple collections from individuals over a range of life stages, especially encompassing periods of organogenesis, would be more useful for determining causative relationships.

Another recent study examined BPA concentrations in urine and blood collected from 516 Korean adults living in urban areas (Hong et al. 2009). Urinary concentrations of total BPA were associated with at least one marker of oxidative stress, although these associations were no longer statistically significant when age, sex, weight, smoking, and exercise were considered in the regression models. Hong et al. (2009) also detected an association between high BPA exposures and increased fasting blood glucose levels, perhaps lending additional weight to a possible link between BPA exposure and diabetes. Another study supports the connection between BPA exposure and oxidative stress; Yang YJ et al. (2009) found that total BPA levels were associated with markers of stress in postmenopausal women, but not in premenopausal women or men, leading the authors to suggest that postmenopausal women may be more susceptible to BPA-induced adverse health effects.

Several smaller studies have examined the effects of BPA exposure on other health outcomes. For instance, BPA levels in blood have been associated with a variety of conditions in women, including obesity, endometrial hyperplasia, endometriosis, recurrent miscarriages, sterility, and PCOS (reviewed by Vandenberg et al. 2007). Other studies detected associations between high BPA exposure and chromosomal abnormalities, including pregnancies with fetuses that had an abnormal karyotype (Yamada et al. 2002), recurrent miscarriage (Sugiura-Ogasawara et al. 2005), and sister chromatid exchange measured in peripheral lymphocytes (Yang et al. 2006). These epidemiological studies have several limitations, including small sample sizes, insufficient information on subject selection criteria, and cross-sectional designs that failed to adequately control for potential confounders (Vandenberg et al. 2007), thus preventing accurate assessments regarding the potential health risks of BPA. Even more recent studies have identified relationships between BPA exposure and reproductive hormone levels in male patients at an infertility clinic (Meeker et al. 2010) and the number of oocytes retrieved from women undergoing IVF fertility treatments (Mok-Lin et al. 2009). Finally, prenatal BPA exposures, as determined by maternal urine concentration during pregnancy and at delivery, were found to be associated with increased externalizing behaviors (i.e., aggression, hyperactivity), especially in female toddlers (Braun et al. 2009).

It is somewhat surprising that most studies undertaken to understand human health hazards have relied heavily on biomonitoring of BPA from the general population, and only a few studies have measured exposures to BPA in occupational settings or have associated occupational exposures with adverse health outcomes. This limitation may stem from the belief that BPA exposure comes predominantly from contamination via food sources. Despite reports suggesting exposure of healthy individuals to BPA by non-oral routes (Stahlhut et al. 2009), a data gap exists relative to exposure levels in employees in BPA-producing industries or those workers using BPA in different manufacturing processes. Thus, measurement strategies and studies to investigate occupational exposure to BPA and health outcomes are very much needed.

## Reliability of Biomonitoring and Toxicokinetic Studies

The need for human biomonitoring of BPA for risk assessment purposes is undisputed because all sources of exposure have not been identified, and thus internal exposures cannot be properly calculated. Groups charged with assessing risk from exposure have heated debates about the toxicokinetics of BPA and the plausibility and reliability of individual data and analytical approaches used in biomonitoring studies. Two studies thus far have attempted to determine the kinetics of BPA metabolism in human subjects (Volkel et al. 2002, 2005).

The first toxicokinetic study was designed to resolve issues regarding the internal exposure to BPA that originated from animal studies: Volkel et al. (2002) administered 5 mg deuterium-labeled BPA (equivalent to 54–90 μg/kg BW) orally to adult volunteers and monitored blood and urinary BPA levels. Unconjugated BPA was always < LODs in both plasma (LOD = 2.28 ng/mL) and urine (LOD = 1.37 ng/mL) at all time points studied after BPA administration; the results were interpreted by the authors as indicative of rapid metabolism, noting that “only a small percentage of the dose of [BPA] is available for other biotransformation pathways, due to the rapid glucuronidation” (Volkel et al. 2002). In support of this statement, concentrations of conjugated BPA were also measured in blood and urine; the authors reported that BPA glucuronide concentrations fell below the LOD in both urine and blood 24–34 hr after BPA administration, although this is not apparent from the concentration–time course model they presented. Using their finding that the terminal half-life of BPA glucuronide in blood was 5.3 hr, Volkel et al. (2002) concluded that conjugated BPA is also rapidly cleared from the blood.

Several inconsistencies in the article by Volkel et al. (2002) raise questions about its reliability. For instance, the authors reported two different values for the time when maximal plasma concentrations (*C*_max_) were achieved (1.35 hr vs. 4 hr). In addition, the BPA glucuronide levels reported in blood are higher than the total BPA concentrations measured in the same individuals. Finally, the authors indicated that they measured BPA metabolism in three women, a group of three men, and then in a separate group of four men, yet the groups of male volunteers clearly overlap (at least 2 men were subjects in both groups), making the data compiled from combining these two groups questionable.

In drawing their conclusion that there is no risk from current human exposure levels, Volkel et al. (2002) overlooked several important points from a risk assessment perspective. First, they did not acknowledge the likelihood of different toxicokinetics when BPA exposure is continuous compared with a single administration. Ginsberg and Rice (2009) suggested that results from the Volkel et al. (2002) study were more consistent with delayed excretion from long-term internal storage or cycling between conjugation and deconjugation. Second, the potential for BPA to have actions at low levels (in the nanograms per milliliter range) was not considered. Third, this toxicokinetic study was designed to assess the metabolism of BPA after oral exposure because, until very recently (Stahlhut et al. 2009), it was assumed that most if not all BPA exposure in humans occurs via the oral route. However, because all sources of BPA have not been identified, non-oral exposures cannot be discounted. Finally, Volkel et al. (2002) overlooked the possibility of differences in toxicokinetics under different physiological paradigms; they used a small mixed-subject group composed of individuals of both sexes and different ages. The differences between sexes and age groups in urinary BPA levels found in CDC biomonitoring studies (Calafat et al. 2005, 2008) raise the possibility that the toxicokinetics of chemicals and drugs, including BPA, are likely to be very different in fetuses and neonates compared with adults. In fact, this assumption was confirmed in two independent kinetic models, which found that the internal exposure to BPA in newborns can be 3–10 times higher than in adults (Edginton and Ritter 2009; Mielke and Gundert-Remy 2009). There is also the potential for deconjugation of BPA glucuronide *in utero* by β-glucuronidase, an enzyme that is present in high concentrations in placenta and various other tissues (Ginsberg and Rice 2009). Furthermore, studies in rodents have found that neonates have limited ability to convert BPA into an inactive conjugated form, independent of the route of administration (Taylor et al. 2008). This may also be true for human fetuses and neonates.

In a follow-up investigation, Volkel et al. (2005) further examined the kinetics of BPA in urine and plasma. They assessed BPA exposure both after administration of labeled BPA and in an environmentally exposed population. For direct testing, subjects were administered 25 μg labeled BPA (equivalent to 0.28–0.43 μg/kg BW), a much smaller dose than that administered in their earlier study; unconjugated and conjugated BPA were then measured in urine (Volkel et al. 2005). Although the authors suggested that they had developed a very sensitive and selective method, the LODs for this study were again higher than those in other studies using the same analytical methods (for unconjugated BPA, LOD = 1.14 ng/mL; for BPA glucuronide, LOD = 10.1 ng/mL).

Volkel et al. (2005) examined BPA kinetics in six individuals administered BPA, although they provided no information about the characteristics of these subjects, making it difficult to draw any conclusions from their study. The authors suggested that there were no differences in kinetics among volunteers, yet a closer look at their results shows a wide variation in BPA measurements across individuals. In the three men examined, 85% of the administered BPA dose was recovered in urine after 5 hr, mostly as BPA glucuronide. In the three women examined, 75% of BPA was recovered as BPA glucuronide after the same period of time. In two of six individuals, unconjugated BPA was detected at levels of approximately 1 ng/mL urine. This finding directly contradicts the conclusions reached by the study authors, who suggested that 100% first-pass metabolism would promptly convert BPA to its conjugated metabolites. As mentioned above, several other studies have also detected unconjugated BPA in urine (Calafat et al. 2009; Kim et al. 2003; Ouichi and Watanabe 2002; Schöringhumer and Cichna-Markl 2007; Volkel et al. 2008; Ye et al. 2005b). Volkel et al. (2005) also collected plasma samples for measurement of BPA, although no measurements of conjugated or unconjugated BPA were reported. Finally, unlike other biomonitoring studies, these investigators failed to detect any BPA in the environmentally exposed group, likely because the LODs in these kinetic studies were 10 to 100 times less sensitive than methods used in most biomonitoring studies. These limited observations emphasize the need for risk assessment studies to employ state-of-the-art analytical techniques that are sensitive enough to detect low levels of BPA with large sample sizes so that one can consider variability in the population and influence from physiological status; these kinetic studies therefore are not appropriate for risk assessment of BPA.

The next issue to consider is whether biomonitoring studies per se are sufficiently reliable and therefore useful for risk assessment purposes. The detection rates and concentrations of BPA in urine and blood of environmentally exposed individuals are remarkably similar in studies performed by several independent groups using state-of-the-art analytical techniques; independent confirmation of these results alone indicates that that these studies are reliable. These findings are further supported by the dozens of additional studies that have used less sensitive or less selective methodologies, because all of these studies report conjugated or unconjugated BPA levels in human samples in a very narrow range of concentrations. Taken together, although the discussions about the reliability of unconjugated BPA measurements within human tissues and fluids appear to be ongoing, especially in the risk assessment community, the consistency of available data on internal exposure to BPA across investigative groups is more than convincing.

Several biomonitoring studies (Calafat et al. 2005, 2008; Kim et al. 2003; Mao et al. 2004) and one of the toxicokinetic studies (Volkel et al. 2005) suggest that men and women differ in their uptake and/or metabolism of BPA. Other studies suggest that pregnant and nonpregnant women differ in their uptake or metabolism of chemicals, including BPA. These findings reinforce the fact that kinetic models based on metabolism of BPA from a mixed group of adults cannot be extrapolated to different physiological states, especially vulnerable populations such as pregnant women and children.

## Utility of Physiologically Based Toxicokinetic Models of BPA

Physiologically based toxicokinetic (PBTK) modeling incorporates information on the physiology and anatomy of the experimental animal or human and the biochemistry of the chemical of interest into a conceptual model for computer simulation. Compared with the essentially empiric kinetic models (i.e., the so-called classical kinetic models), PBTK modeling has the advantage of being more grounded in physiology. PBTK models are powerful tools for interpolations and extrapolations that are particularly useful in the context of risk assessment, such as dose to dose, route to route, single to multiple exposure, continuous to discontinuous exposure, species to species, “external” or administered dose and internal or target tissue concentration, males to females, adults to children, nonpregnant women to pregnant, and so on (Loizou et al. 2008).

A few PBTK models have been developed for describing the toxicokinetics of BPA in rats (Shin et al. 2004), pregnant mice (Kawamoto et al. 2007), adult humans (Teeguarden et al. 2005), and children < 2 years of age (Edginton and Ritter 2009). First, Shin et al. (2004) developed a PBTK model involving vein, artery, lung, liver, spleen, kidneys, heart, testes, muscle, brain, adipose tissue, and small intestines for predicting tissue distribution and kinetics of BPA in rats. The model was validated by predicting the steady-state levels of BPA in the blood and tissues of rats that had received repeated intravenous injections. These investigators used their PBTK model to simulate blood and tissue levels of unconjugated BPA in an adult human (70 kg BW) receiving multiple oral doses of BPA (100 mg every 24 hr for 10 days) and found steady-state venous blood levels of 1.3 ng/mL, a concentration remarkably similar to what is measured in environmentally exposed humans (Table 3).

In a model of oral route exposure to BPA in rats and humans, Teeguarden et al. (2005) imposed restrictions on the concentration of unbound (free) BPA resulting from plasma protein binding and predicted the degree of ER binding that may occur in the rat uterus at different BPA doses. Based on simulations using the model, terminal BPA elimination in rats, but not humans, was found to be strongly influenced by the enterohepatic recirculation of BPA glucuronide. The oral route blood kinetics in rats and the oral route plasma and urinary elimination kinetics in humans described by the model of Teeguarden et al. (2005) were consistent with data reported by Pottenger et al. (2000) and Volkel et al. (2002) for BPA-treated rats and human volunteers, respectively. However, their model was based on single exposures to BPA and did not account for repeated exposures to humans, as actually occurs. Nonetheless, as far as toxicokinetic modeling in humans is concerned, plasma levels of unconjugated BPA predicted by the physiologically based pharmacokinetic (PBTK) model for a single oral dose of 5 mg BPA per person were less than the analytical LOD of the method used by Volkel et al. (2002). Thus, only appropriately sensitive analytical methods should be used to determine metabolic parameters after administration of BPA.

More recently, a PBTK model was developed to assess the age dependence of the toxicokinetics of both unconjugated BPA and BPA glucuronide in children < 2 years of age (Edginton and Ritter 2009). This PBTK model was initially built using information gathered from toxicokinetic studies of BPA in adults and then scaled down taking into account the age dependence of physiological parameters relevant for absorption, distribution, metabolism, and elimination. According to the model predictions, steady-state plasma concentrations of unconjugated BPA in newborns were expected to be 11 times higher than those found in adults who received the same body weight–normalized dose. Taking into account estimates of dietary exposures, plasma levels of unconjugated BPA in 3- to 6-month-old children were expected to be 5 times higher than the levels found in adults. Simulations using this PBTK model are therefore consistent with the idea that newborns and young children are internally exposed to higher levels of unconjugated BPA than levels that have been estimated for adults. Indeed, data from infants in neonatal infant care units in American hospitals suggested that BPA levels in these infants were 11 times higher than GM concentrations in American adults (Calafat et al. 2009), matching exactly the predictions from the PBTK model.

Another PBTK model—one for BPA in pregnant mice—was developed by Kawamoto et al. (2007). The authors used experimental kinetic data from a single oral dose of BPA administered to pregnant mice on gestational day 15 for calibrating their kinetic model. Simulations using their PBTK model indicated that (total) BPA was rapidly transferred through the placenta to the fetus and that it was only slowly eliminated from the fetal compartment. It should be stressed that this model was calibrated using data for total (unconjugated plus conjugated) BPA, so it did not specifically estimate the toxicokinetics of unconjugated BPA. As far as we are aware, no other PBTK model for BPA in pregnant rodents or pregnant humans has been constructed. Additional and more refined PBTK models of unconjugated BPA in pregnant rodents and humans are therefore urgently needed.

Current PBTK models for BPA do not take into consideration the influence of local deconjugation of BPA metabolites at or in the vicinity of target tissues. Ginsberg and Rice (2009) recently discussed literature reports supporting the biological plausibility of local deglucuronidation of BPA glucuronide occurring at a number of target tissues, such as placenta and the fetal compartment. For instance, Takahashi and Oishi (2000) demonstrated that placenta has rather extensive β-glucuronidase activity and that liver and kidneys show much higher unconjugated BPA levels than blood. More information is needed to determine if, in fact, BPA is deconjugated *in situ*; if so, this should be added to future models.

Finally, a recent study determined that there is a linear relationship between applied doses and circulating unconjugated BPA concentrations (Vandenberg et al. 2007). This linear relationship was used to back-calculate the applied dose needed to generate the concentrations of unconjugated BPA that have been repeatedly measured in environmentally exposed humans; the applied dose needed to achieve circulating BPA levels of 1–3 ng/mL blood that have been detected in biomonitoring studies (Table 3) exceeded 500 μg/kg/day. This matches the predictions from the PBTK model developed by Shin et al. (2004), which clearly indicated that doses of 100 mg every 24 hr for 10 days were needed to produce blood levels of 1.3 ng/mL in rats. These data need to be verified, but if true, they provide strong evidence that one cannot use urine levels to back-calculate to exposure levels.

## Future Directions and Research Needs

Throughout this review we have mentioned research needs; these are summarized below.

We suggest that several small biomonitoring studies performed previously should be repeated using large reference populations. First, it is imperative that estimates of urinary BPA levels over 24 hr be undertaken. Second, there is a need for obtaining multiple samples of blood and urine from individuals to assess variability of exposure over time, likely encompassing weeks, months, or years. Finally, large-scale biomonitoring studies across the life span are needed to confirm levels of total and unconjugated BPA in blood. These studies need to compare exposure levels in men and women, adults, neonates, children (spanning the prepubertal to pubertal period), pregnant and nonpregnant women, and obese and nonobese individuals, and across disease states. It is clear from animal studies that developmental periods (*in utero* and neonatal periods) are the most sensitive to BPA; thus, special attention should be given to assessment of total and unconjugated BPA in these vulnerable populations.

More studies are needed to examine pregnancy outcomes from large groups of racially and socioeconomically diverse women, relating them to internal exposure levels throughout pregnancy. It has been hypothesized that racial disparities in exposures to BPA and other endocrine-disrupting chemicals may account for the reduced fetal survival and birth weight of offspring (Ranjit et al. 2010). Recent epidemiology studies suggest relationships between urinary BPA concentrations and fertility end points (Meeker et al. 2010; Mok-Lin et al. 2009). Additional studies are needed to extend these findings and also to define the levels of total and unconjugated BPA in ovarian follicular fluid, semen, amniotic fluid, and cord blood to relate to reproductive disease outcomes and adult consequences.

Human toxicokinetic studies of BPA that employ sensitive methods across physiological states and age groups are needed. In addition, studies directly comparing the toxicokinetics of BPA metabolism in humans and laboratory animals will help to determine if animals can accurately predict human metabolism.

Studies are needed to identify all sources of exposure and to assess the daily dose of BPA actually coming from the oral route versus other routes. Measurement of BPA in all food and drink sources and in indoor and outdoor air samples could be mandated by legislative actions.

Longitudinal studies in children linking developmental exposures to BPA and later-onset diseases are especially important and needed. Two epidemiological studies have related adult urinary BPA concentrations to disease outcomes such as obesity, type 2 diabetes, and cardiovascular disease (Lang et al. 2008; Melzer et al. 2010), all of which are on the rise. Studies that focus on relating BPA exposure to disease outcomes should also control for dietary phytoestrogen exposure and consider mixture effects.

In addition to these specific data needs, it is important to take advantage of the NHANES data and database (http://www.cdc.gov/nchs/nhanes.htm) for assessment of BPA levels and the relationship of those levels and disease outcomes. Several recent studies are excellent examples of using this database to answer important questions relating to BPA exposure and linking BPA exposures to disease outcomes (Lang et al. 2008; Stahlhut et al. 2009). The NHANES database is a virtual treasure of data awaiting analysis and comparison.

## Conclusions

We believe that human biomonitoring data clearly indicate that the general population is exposed to BPA ubiquitously, including significant internal exposures to unconjugated BPA. More important, animal studies suggest that fetuses and children are particularly vulnerable to BPA exposures and, at the same time, are exposed to higher levels of unconjugated BPA.

The two toxicokinetic studies performed to date (Volkel et al. 2002, 2005), which suggest that human exposure is negligible, have significant flaws and are therefore not reliable for risk assessment purposes. Further, the biomonitoring data, coupled with predictions from PBTK models, indicate that human exposures are higher than have been suggested from the toxicokinetic studies. Weighing all the evidence available to date—because of the significant data showing human exposures to unconjugated BPA and animal data indicating increased susceptibility to disease at levels found in humans—we recommend that the precautionary principle be followed until further data are available on exposure of fetuses and children to BPA. The health of the public is at stake.

## Figures and Tables

**Table 1:** BPA levels in human urine after environmental exposures.

Reference	Detection method and enzymatic treatment^a^	LOD (ng/mL)	Sample size	Study population	Detection rate	BPA level [ng/mL (ppb), mean ± SE]	
						Unconjugated BPA	Total BPA
Brock et al. 2001	GC-MS^b^	0.12	Five pools	Each pool contained ≥ 5 individuals	5/5 pools	All < LOD	Range, 0.11–0.51

Ouchi and Watanabe 2002	HPLC-ECD with column switching^b^	0.2	48	Female college students (Japan)	2% unconjugated BPA, 100% BPA glucuronide	Range, ND–0.2^c^	

Kim et al. 2003	RP-HPLC–FD	0.28	15	Korean males	100% unconjugated and total BPA	0.58 ± 0.14^c^,^d^	2.82 ± 0.73
			15	Korean females		0.56 ± 0.10^c^,^d^	2.76 ± 0.54

Matsumoto et al. 2003	HPLC	1.7	50	College students in 1992	82%		Exact values NR
			56	College students in 1999	61%		Exact values NR

Tsukioka et al. 2003	NCI–GC-MS^b^	0.1	6	Japanese adults	100%		Mean, 1.6

Yang et al. 2003	HPLC-FD^b^	0.012	73	Koreans with various *SULT1A1* polymorphisms	75%		Mean, ~ 9.5

Arakawa et al. 2004	GC-MS/MS^b^	0.38	36	Japanese males	100%		Range, 0.2–14 (μg/day)

Mao et al. 2004	HPLC-FD^e^	2.7	10	Chinese males	60%		Range, ND–395
			10	Chinese females	100%		Range, 3–374

Calafat et al. 2005	GC-MS^b^	0.1	184	American males	96%		GM, 1.63
			210	American females	94%		GM, 1.12

Kawaguichi et al. 2005	SBSE–GC-MS	0.02	5	Adult volunteers	80%		Range, ND–5.41

Liu et al. 2005	HPLC with ECD^b^	0.5	9	American girls, 9 years of age	89%		Median, 2.4
			24	American adults	52%		Median, 0.47

Volkel et al. 2005	HPLC-MS/MS^b^	1.14	6	German adults	0%	—c	< LOD

Ye et al. 2005a	Online SPE-HPLC-MS/MS	0.4	30	American adults	87%		GM, 3.5

Ye et al. 2005b	Online SPE-HPLC-MS/MS	0.3	30	American adults	97%	Mean, < LOD^c^,^d^	Mean, 3.2

Martin et al. 2005	SPE-HPLC-MS/MS^b^	NR	19	Children before dental treatment, ages not given	NR		GM, 0.26

Joskow et al. 2006	GC-MS^b^	0.1	14	Men before dental treatment	NR		2.41 ± 0.33

Yang et al. 2006	HPLC-FD^b^	0.026	172	Koreans with various *SULT1A1* polymorphisms	97.5%		Median, 7.86

Moors et al. 2007	GC-MS	3	15	German adults	60%		Range, ND–55

Schöringhumer and Cichna-Markl 2007	SGIC-HPLC-FD	0.2	12	Austrian adults	75% unconjugated BPA, 100% total	Median, 0.3	Median, 1.1
			10	Dialysis patients (Austria)	90% unconjugated BPA, 100% total	Median, 0.2	Median, 1.2

Wolff et al. 2007	HPLC-MS/MS^b^	0.36	90	American girls, 6–9 years of age	94%		GM, 2.0

Calafat et al. 2008	HPLC-MS/MS	0.4	314	Children, 6–11 years of age	92.6% of all individuals examined		GM, 4.3
			713	Adolescents, 12–19 years of age			GM, 2.8
			950	Adults, 20–59 years of age			GM, 2.4
			537	Adults ≥ 60 years of age			GM, 2.3

García-Prieto et al. 2008	CME-LC-FD^b^	0.197	8	Spanish college students	25% unconjugated BPA, 100% total		Range, 4.03–49

Mahalingaiah et al. 2008	HPLC-MS/MS	0.4	45	Women in a fertility clinic	87% of all samples		Range, ND–42.6; GM, 1.09
			37	Men in a fertility clinic			Range, ND–18.7; GM, 1.62

Teitelbaum et al. 2008	HPLC-MS/MS	0.36	159 samples from 35 children	American children, 6–10 years of age	95%		GM, 3.4

Volkel et al. 2008	Online SPE-HPLC-MS/MS^b^	0.3	31	German women	NR for any samples or groups	ND–2.5	ND–6.5
			30	German children, 5–6 years of age		ND–0.9	ND–7.5
			315 samples from 203 subjects	Archived samples collected from adults (2005)		All < LOQ (5 ng/mL)	NR
			62 samples from 21 subjects	Laboratory workers (Germany)		ND–1.8	ND–3.3

Wolff et al. 2008	Online SPE-HPLC-MS/MS	0.36	404	Pregnant American women	90.8%		Range, ND–35.2

Ye et al. 2008b	GC-MS/MS^b^	0.26	100	Pregnant Dutch women	82%		Median, 1.2

Becker et al. 2009	LC/LC-MS/MS	0.25	137	German children, 3–5 years of age	99% of all samples > LOQ		GM, 3.55
			145	German children, 6–8 years of age			GM, 2.72
			149	German children, 9–11 years of age			GM, 2.22
			168	German adolescents, 12–14 years of age			GM, 2.42

Calafat et al. 2009	Online SPE-HPLC-MS/MS	0.4	41^g^	Premature American infants	92% unconjugated BPA, 100% total	Range, ND–17.3; median, 1.7 GM, 1.8	Range, 1.6–946; median 28.6, GM, 30.3

Carwile et al. 2009	Online SPE-HPLC-MS/MS	0.4	77	American college students avoiding polycarbonate bottles	88%		GM, 1.3
				Same students using polycarbonate bottles	96%		GM, 2.1

He et al. 2009	HPLC^b^	0.31	419	Chinese males	58%		GM, 1.41
			503	Chinese females	44%		GM, 0.58

Hong et al. 2009	HPLC-MS/MS	0.063	516	Korean adults	76%		2.742 ± 0.39

Nepomnaschy et al. 2009	HPLC-MS/MS^b^	0.18	60	Premenopausal women	100%		1.82 ± 0.33

Yang YJ et al. 2009	HPLC-MS/MS	0.0063	259	Korean men	75%		GM, 0.52
			92	Premenopausal Korean women	80%		GM, 0.61
			134	Postmenopausal Korean women	75%		GM, 0.58

Ye et al. 2009a	HPLC-MS/MS	0.26	10 pools	Pregnant Norwegian women, 110 total	NR for any samples or groups		GM, 4.50
			110	Pregnant Dutch women			GM, 2.52
			87	Pregnant American women			GM, 3.93

Abbreviations: CME, coacervative microextraction; ECD, electron capture detection; FD, fluoremetric detection; GM, geometric mean; LOQ, limit of quantification; NCI, negative chemical ionization; ND, not detected; NR, not reported; RP, reverse phase; SBSE, stir bar sorptive extraction; SGIC, sol-gel immunoaffinity column; SPE, solid-phase extraction; *SULT1A1*, sulfotransferase 1A1 gene.

a All samples were treated with glucuronidase and sulfatase unless otherwise noted.

b Samples were treated with glucuronidase only.

c BPA glucuronide was also measured.

d BPA sulfate was also measured.

e Samples were treated with chemical hydrolysis, which deconjugates both sulfate and glucuronide groups.

f Corrected for creatinine and presented as μg/g creatinine.

g Only 37 samples were tested for unconjugated BPA.

**Table 2:** BPA levels in saliva.

Reference	Detection method	LOD (ng/mL)	Sample size	Sample	End point(s)	Leaching level (μg/mL)
Olea et al. 1996	HPLC (verified by GC-MS)	NR	18	Patients with 50 mg of sealant applied to a total of 12 molars	Saliva 1 hr after application	Range, 3.3–30

Arenholt-Bindslev et al. 1999	HPLC	100	8	Patients with a total of 38 mg of sealant applied to a total of 4 molars	Saliva immediately after application	Range, ND–2.8; mean, 1.43
					Saliva 1 hr after application	Undetected in any sample
					Saliva 24 hr after application	Undetected in any sample

Fung et al. 2000	HPLC-FD	5	22	Patients with 32 mg of sealants applied to a total of 4 molars	Saliva 1–3 hr after application	Range, 0.0058–0.1056

Zafra et al. 2002	GC-MS	3	8	Patients undergoing dental repairs	Saliva 1 hr after application	Range, 0.0153–0.0324

Sasaki et al. 2005	ELISA	NR	21	Patients treated with one of nine resins	Saliva immediately after application	Range, 0.0210–0.0601
					Saliva after application and gargling	Range, 0.0016–0.0047

Joskow et al. 2006	GC-MS	0.1	14	Patients treated with one of two dental sealants (Delton and Helioseal)	Saliva before dental sealant application	0.00030 ± 0.000043^a^
					Saliva immediately after Delton sealant application	0.0428 ± 0.01032
					Saliva 1 hr after Delton sealant application	0.00786 ± 0.00424
					Saliva immediately after Helioseal sealant application	0.00054 ± 0.00020
					Saliva 1 hr after Helioseal sealant application	0.00021 ± 0.000013

Abbreviations: FD, fluoremetric detection; ND, not detected; NR, not reported.

a Values are mean ± SE.

**Table 3:** BPA levels in human serum/blood after environmental exposures (nonpregnant adults).

Reference	Detection method	LOD (ng/mL)	Sample size	Sample type	Subject description	BPA level^a^ (ng/mL, mean ± SE)	Other individuals examined
Sajiki et al. 1999	Electrochemical detection or MS-ESI	0.1–0.2	12	Serum	Healthy Japanese women	Range, 0–1.6; 0.33 ± 0.54	
			9		Healthy Japanese men	Range 0.38–1.0; 0.59 ± 0.21	

Fung et al. 2000	HPLC-FD	5	40	Blood	Healthy American volunteers before dental sealant application	ND	Individuals after dental sealant application^b^

Inoue et al. 2000	HPLC with electrochemical detection	0.01 in solvent	6	Serum	Healthy Japanese adults	Mean 0.32	
	Coulometric array	0.05 in serum					

Inoue et al. 2001	LC-MS	0.1	3	Blood or plasma^c^	Healthy Japanese adults	ND–1.0	

Ikezuki et al. 2002	ELISA	0.3 in serum	30	Serum	Healthy Japanese women	2.0 ± 0.146	Pregnant women, fetuses^d^

Takeuchi and Tsutsumi 2002	ELISA	0.3 in serum	11	Serum	Healthy Japanese men	1.49 ± 0.11	Women with PCOS^e^
			14	Serum	Healthy Japanese women	0.64 ± 0.1	

Yoshimura et al. 2002	GC-MS with NCI	5 pg/mL	20	Pooled serum (≥ 5 individuals per pool)	NR	0.54 ± 0.037	

Kuroda et al. 2003	HPLC fluorescence derivation, column switching	0.04	21	Serum	Sterile Japanese women	0.46 ± 0.044	Pregnant women, fetuses^d^

Hiroi et al. 2004	ELISA	0.5 (from Kodaira et al. 2000)	11	Serum	Healthy Japanese women	2.5 ± 0.452	Women with endometrial hyperplasias and cancer^f^

Takeuchi et al. 2004	ELISA	0.3 in serum	19	Serum	Healthy Japanese women	0.71 ± 0.09	Women with obesity, PCOS, or both^g^

Sugiura-Ogasawara et al. 2005	ELISA	0.5 (from Kodaira et al. 2000)	32	Serum	Healthy Japanese women	0.77 ± 0.067	Women with recurrent miscarriage^h^

Volkel et al. 2005	LC-MS/MS	0.57–1.14	19	Plasma	NR	ND	

Liu et al. 2007	LC-DAD-MS	0.05	10	Serum	Healthy Chinese adults	ND–0.28	

Dirtu et al. 2008	SPE-GC-ECNI-MS	0.003	7	Individual serum samples	Healthy Belgian adults	0.98 ± 1.09	
			14	Pooled serum samples	Healthy Belgian women	1.17 ± 1.09	

He et al. 2009	HPLC	0.39	404	Serum	Healthy Chinese men	GM, 0.20	
			482	Serum	Healthy Chinese women	GM, 0.16	

Kaddar et al. 2009	RIA	0.08	207	Plasma	French hospital patients, unknown health status	ND to > 2, detected in 83%	Patients with regular dialysis treatment^i^

Yang M et al. 2009	HPLC-FD	0.012	82	Blood	Healthy Korean women	Median, 0.03	Breast cancer patients^j^

Abbreviations: DAD, photodiode array detection; ECNI, electron capture negative ionization; ESI, electrospray ionization; FD, fluorometric detection; GM. geometric mean; NCI, negative chemical ionization; NR, not reported; ND, not detected; SPE, solid-phase extraction.

a In studies where two populations were compared, concentrations reported are for healthy controls only.

b Unconjugated BPA was not detected after application of dental sealants.

c The authors interchange “blood” and “plasma,” so it is difficult to determine which was actually assessed.

d See Table 4.

e Mean concentration in women with PCOS, 1.04 ± 0.1 ng/mL.

f Mean concentration in women with simple endometrial hyperplasias (benign), 2.9 ± 0.632 ng/mL; women with complex endometrial hyperplasias (malignant potential), 1.4 ± 0.133 ng/mL; women with postmenopausal endometrial cancer, 1.4 ± 0.189 ng/mL.

g Mean concentration in women with obesity (no PCOS), 1.04 ± 0.09 ng/mL; PCOS (no obesity), 1.05 ± 0.10 ng/mL; obesity and PCOS, 1.07 ± 0.16 ng/mL.

h Mean concentration in women with recurrent miscarriage, 2.59 ± 0.780 ng/mL.

i Mean concentration not provided for dialysis patients; > 70% had > 10 ng/mL.

j Median concentration for breast cancer patients, 0.61 ng/mL.

**Table 4:** BPA levels in human serum and blood during pregnancy (maternal) or gestation (fetal).

Reference	Detection method	LOD (ng/mL)	Sample size	Sample type	Study population	BPA level [ng/mL (ppb), mean ± SE]
Ikezuki et al. 2002	ELISA	0.3 (serum)	37	Maternal serum^a^	Japanese women in early pregnancy	1.5 ± 0.197
			37	Maternal serum	Japanese women in late pregnancy	1.4 ± 0.148
			32	Fetal (cord) serum		2.2 ± 0.318

Schönfelder et al. 2002	Derivatization-GC/MS	0.01 (serum)	37	Fetal (cord) serum	German women at delivery	2.9 ± 0.411
			37	Maternal serum		4.4 ± 0.641

Yamada et al. 2002	ELISA	0.5	200	Maternal serum	Japanese women carrying fetuses with normal karyotypes	Median, 2.24
			48	Maternal serum	Japanese women carrying fetuses with abnormal karyotypes	Median, 2.97

Kuroda et al. 2003	HPLC fluorescence derivation, column switching	0.04	9	Maternal serum^a^	Japanese women at delivery	0.46 ± 0.067
			9	Fetal cord serum		0.62 ± 0.043

Tan and Mohd 2003	GC-MS	0.05	180	Fetal cord plasma	Samples collected in Malaysia	Range, ND–4.05 (88% with positive detection)

Lee et al. 2008	HPLC-GC-MS	0.625	300	Maternal blood	Korean women at delivery	9.04 ± 0.81 Range, ND–66.48
			300	Fetal (cord) blood		1.13 ± 0.08 Range, ND–8.86

Padmanabhan et al. 2008	HPLC-ESI-MS/MS	0.5	40	Maternal blood	American women at delivery	5.9 ± 0.94 Range, ND–22.3

Abbreviations: ESI, electrospray ionization; ND, not detected.

a Data on nonpregnant females is included in Table 3.

**Table 5:** BPA levels in human tissues and fluids during pregnancy and lactation.

Reference	Detection method	LOD (ng/mL)	Sample size	End point(s)	Study population	BPA level [ng/mL (ppb), mean ± SE]
Ikezuki et al. 2002	ELISA	0.3	32	Early amniotic fluid (15–18 weeks)	Pregnant Japanese women	8.3 ± 1.573
			38	Late amniotic fluid (at full term, before delivery)	Pregnant Japanese women undergoing cesarian section	1.1 ± 0.162
			36	Follicular fluid	Japanese women undergoing IVF procedures	2.4 ± 0.133

Schönfelder et al. 2002	Derivatization-GC-MS	0.01	37	Placenta	German women at delivery	11.2 ± 1.512 ng/g

Todaka and Mori 2002	GC-MS	NR	NR	Umbilical cord tissue at birth	Samples collected in Japan	Mean, 4.4 ± 1.5 ng/g; range, 0.11–15.2 ng/g

Yamada et al. 2002	ELISA	0.5	200	Normal fetal amniotic fluid (14–18 weeks)	Pregnant Japanese women	Median, 0.26; range, ND–5.62
			48	Abnormal fetal karyotype fetal amniotic fluid (14–18 weeks)	Pregnant Japanese women	Median, 0

Otaka et al. 2003	SPE-GC-MS	0.09	3	Breast milk	Japanese women	Range, ND–0.70 ng/g

Sun et al. 2004	DIB-Cl derivatization-HPLC	0.11	23	Breast milk	Japanese women	0.61 ± 0.042

Engel et al. 2006	HPLC-electrochemical detection	0.5	21	Residual amniotic fluid from amniocentesis, < 20 weeks’ gestation	American women > 35 years of age	Mean, 0.55 (10% > 0.5 ng/mL)

Ye et al. 2006	Online SPE-HPLC-MS/MS	0.28	20	Breast milk	American women	Unconjugated BPA: mean, 1.3; 60% detection Total BPA: mean, 1.9; 90% detection

Kuruto-Niwa et al. 2007	ELISA	0.3	101	Human colostrum	Japanese women 3 days after delivery	3.41 ± 0.013

Ye et al. 2008a	Online SPE-HPLC-MS/MS	0.3	4	Breast milk	American women	Unconjugated BPA: mean, 0.80 Total BPA: mean, 1.02

Kaddar et al. 2009	RIA	0.08	17	Follicular fluid	French women undergoing IVF procedures	Range, ND–1.0; 39% detection

Abbreviations: DIB-Cl, 4-(4,5-diphenyl-1H-imidazol-2-yl)benzoyl chloride; ND, not detected; SPE, solid-phase extraction.
